# Genetic susceptibility to optic neuropathy in patients with alcohol use disorder

**DOI:** 10.1186/s12967-024-05334-0

**Published:** 2024-05-25

**Authors:** Camille Delibes, Marc Ferré, Marine Rozet, Valérie Desquiret-Dumas, Alexis Descatha, Bénédicte Gohier, Philippe Gohier, Patrizia Amati-Bonneau, Dan Milea, Pascal Reynier

**Affiliations:** 1 Département d’Ophtalmologie, Centre Hospitalier Universitaire (CHU), 49000 Angers, France; 2 Université d’Angers, Unité Mixte de Recherche (UMR) MITOVASC, Institut National de la Santé et de la Recherche Médicale (INSERM U-1083), Centre National de la Recherche Scientifique (CNRS 6015), 49000 Angers, France; 3 Département de Biochimie et Biologie Moléculaire, Centre Hospitalier Universitaire, 49000 Angers, France; 4 Département de Psychiatrie et d’Addictologie, Centre Hospitalier Universitaire, 49000 Angers, France; 5 Univ. Angers (University of Angers), CHU Angers, Univ. Rennes, Inserm, EHESP, IRSET (Institut de Recherche en Santé, Environnement et Travail) - UMR_S 1085, IRSET-ESTER, SFR ICAT, CAPTV CDC, 49000 Angers, France; 6 Department of Occupational Medicine, Epidemiology and Prevention, Donald and Barbara Zucker School of Medicine, Hosftra University Northwell Health, New York, NY 11021 USA; 7 Univ Angers, Université de Nantes, LPPL, SFR CONFLUENCES, 49000 Angers, France; 8 Singapore National Eye Centre, Singapore Eye Research Institute, Duke-NUS, Singapore, Singapore; 9 Rothschild Foundation Hospital, Paris, France

**Keywords:** Alcohol use disorder, Ganglion cell complex, Hereditary optic neuropathy, Mitochondria, Mitochondrial DNA, Optic nerve, Optical coherence tomography, Toxic-nutritional optic neuropathy, Retinal nerve fiber layer

## Abstract

**Background:**

The pathophysiology of toxico-nutritional optic neuropathies remains debated, with no clear understanding of the respective roles played by the direct alcohol toxicity, smoking and the often associated vitamin deficiencies, which are risk factors for optic neuropathy. Our aim was to investigate genetic susceptibility in patients with bilateral infraclinical optic neuropathy associated with chronic alcohol use disorder.

**Methods:**

This retrospective cohort study included 102 visually asymptomatic patients with documented alcohol use disorder from a French reference center. Optic neuropathy was identified with optical coherence tomography (OCT), after which genetic susceptibility in the group of affected patients was investigated. Genetic testing was performed using panel sequencing of 87 nuclear genes and complete mitochondrial DNA sequencing.

**Results:**

Optic neuropathy was detected in 36% (37/102) of the included patients. Genetic testing of affected patients disclosed two patients (2/30, 6.7%) with optic neuropathy associated with pathogenic variants affecting the *SPG7* gene and five patients (5/30, 16.7%) who harbored variants of uncertain significance close to probable pathogenicity in the genes *WFS1*, *LOXL1*, *MMP19*, *NR2F1* and *PMPCA*. No pathogenic mitochondrial DNA variants were found in this group.

**Conclusions:**

OCT can detect presence of asymptomatic optic neuropathy in patients with chronic alcohol use disorder. Furthermore, genetic susceptibility to optic neuropathy in this setting is found in almost a quarter of affected patients. Further studies may clarify the role of preventative measures in patients who might be predisposed to avoidable visual loss and blindness.

**Supplementary Information:**

The online version contains supplementary material available at 10.1186/s12967-024-05334-0.

## Introduction

Optic nerve damage associated with alcohol consumption was described at the end of the nineteenth century, giving rise to the concept of “tobacco-alcohol amblyopia” [1]. The pathophysiology of this disorder is still the subject of debate and there is no clear understanding of the respective roles played by the direct alcohol toxicity, smoking and the often associated nutritional disorders, which are risk factors for optic neuropathy [2, 3]. Similar phenotypes of optic neuropathy were reported in association with severe malnutrition during the Second World War, in prisoners of war in Vietnam, and, more recently, after prolonged vegan diets without vitamin supplementation [4, 5]. The optic neuropathy epidemic in Cuba (1991–1993) is an example of the intertwining of the different mechanisms involved, i.e., deficiencies of both folate and B12, as well as exposure to cyanide and methanol [6, 7]. For these reasons and because visual recovery may occur after cessation of alcohol consumption and vitamin B supplementation, the term “tobacco-alcohol amblyopia” has been replaced by the term “nutritional optic neuropathy”, suggesting that nutritional factors may play a preponderant role in its pathophysiology. Surprisingly, however, optic neuropathy can occur in this setting even in the absence of a proven vitamin deficiency [8, 9].

Clinically, patients with this potentially blinding optic neuropathy present with bilateral, progressive, and painless visual loss, red-green dyschromatopsia, and central or caeco-central scotomata. At early stages, the fundus examination is typically normal, but over time, the optic nerve heads become bilaterally pale, eventually progressing to optic nerve atrophy. Visual evoked potentials show conduction damage in the optic nerves, with a decrease in amplitude and/or an increase in latency of the P100 wave [10]. Its diagnosis requires prior exclusion of other causes, including optic nerve compression, inflammation, infection and drug toxicity (ethambutol, disulfiram, etc.).

The hypothesis, according to which reactive oxygen species accumulates at the origin of an oxidative stress, leading to retinal ganglion cell apoptosis, has recently been evoked to describe a common mechanism for the diverse causes of optic neuropathies, including those related to alcohol toxicity [11]. This is consistent with the fact that smoking and heavy alcohol intake might be associated factors in patients with Leber’s hereditary optic neuropathy (LHON) due to mitochondrial DNA mutations [12]. Recent data also suggest that alcohol and tobacco intoxication is more frequent in symptomatic LHON patients, compared to both asymptomatic carriers and the general population [13]. As LHON and tobacco-alcohol amblyopia share common features, it has been proposed that patients with the latter phenotype should be investigated for LHON mutations [14, 15]. Lastly, it has been recently reported that increased alcohol consumption during lock-down periods of Covid-19 has precipitated the development of severe optic neuropathy in patients with LHON variants [16]. Taken together, these arguments suggest that alcohol might be an important environmental factor that might affect the penetrance of LHON. In summary, the co-occurrence of alcohol intoxication and LHON raises the question of a more general genetic contribution to the toxico-nutritional optic neuropathies.

The objectives of this study were (1) to estimate the prevalence of retinal neuronal and axonal loss as measured with optical coherence tomography (OCT) in patients with documented alcohol use disorder (AUD); and (2) to investigate a possible genetic susceptibility to optic neuropathy in this group of patients, using routine sequencing panels of nuclear and mitochondrial genes responsible for hereditary optic neuropathies.

## Materials and methods

### Ethics statement

This retrospective study was carried out in accordance with the ethical recommendations of the Declaration of Helsinki. It obtained a favorable opinion from the Ethics Committee of the University Hospital of Angers, France, under the reference 2020/149. Data from the routine care of patients followed for alcohol intoxication were collected and retrospectively analyzed. The genetic analyses, carried out as part of routine etiological diagnosis of optic neuropathy, were performed after obtaining patients’ informed consent.

### Study participants

We collected health data from 102 patients consecutively enrolled over six months (between May and November 2019), in a dedicated AUD unit in the Psychiatry and Addictology Department of the University Hospital Center of Angers, France. All included patients (age 21.5–62.3 years) were diagnosed with AUD according to the diagnostic criteria of the Diagnostic and Statistical Manual of Mental Disorders, 5th edition (DSM-5). None of the included patients reported visual symptoms. Their optic nerve structure was, however, systematically evaluated, using OCT in order to detect retinal neuronal and axonal loss, suggestive of an infraclinical optic neuropathy. Patients with previously known or concurrent associated retinopathies or optic neuropathies were excluded from the study.

Ophtalmic investigations used OCT imaging centered on the optic discs (Topcon DRI Triton Swept-Source OCT), aiming to measure the thickness of both the ganglion cell complex (GCC) and the peripapillary retinal fiber layer (RNFL). As age leads to a reduction in the amount of GCC, an adjustment was made by the device using linear regression. Depending on the OCT findings, included patients were divided into two groups: (1) a group of patients with retinal neuronal loss, defined as thinning of the average GCC in at least one eye, compared to the normative data; and (2) a group of patients without retinal neuronal loss, if the OCT results were within normal limits in both eyes. All patients in the “optic neuropathy group” underwent a complete clinical ophthalmic examination, including evaluation of the best corrected visual acuity, intraocular pressure and a formal visual field. Color vision was evaluated using a 15 Hue desaturated color vision test. All the patients with retinal neuronal loss underwent an extensive work-up, aimed to rule out other causes of optic neuropathies (i.e., compressive, inflammatory, infectious, glaucomatous, etc.). Included patients underwent measurements of plasmatic vitamins B9, B12, B1 and B6 levels.

### Molecular analyses

The search for genetic susceptibility variants was carried out in the Department of Biochemistry and Molecular Biology of the University Hospital Center of Angers, France. The genetic analysis included complete sequencing of the mitochondrial DNA and a panel of 87 nuclear genes, routinely used for the diagnosis of hereditary optic neuropathies [17].

Genomic DNA of patients was extracted from peripheral blood using a DNA blood kit on an EZ1 apparatus (Qiagen, Courtaboeuf, France). The presence of LHON mutations and other pathogenic variants in the mitochondrial DNA was assessed using complete mtDNA sequencing, as previously described [18]. The panel of 87 nuclear genes included the following known and candidate genes involved in hereditary optic neuropathies: *ACO2, ACOX1, AFG3L2, AGXT, ALG3, ANTXR1, AOX1, ASPA, ATAD3A, ATP1A3, ATXN1, ATXN7, AUH, BOLA3, BTD, C12orf65, C19orf12, CCDC88A, CISD2, CYP1B1, CYP7B1, DNAJC19, DNM1L, DNMT1, DPYD, FA2H, FDXR, FH, FIS1, HSD17B10, IBA57, KLC2, LOXL1, LTBP2, MECR, MFF, MFN1, MFN2, MIEF1, MIEF2, MMP19, MTPAP, MYOC, NDUFS2, NEFH, NMNAT1, NR2F1, OMA1, OPA1, OPA3, OPN1SW, OPTN, PCLO, PDHX, PDSS1, PLAA, PLP1, PMPCA, POLR3A, PRPS1, RAB3GAP1, RPIA, RTN4IP1, SIX6, SLC19A2, SLC25A46, SLC52A2, SON, SPG7, SSBP1, TBCD, TBCE, TFG, TIMM50, TIMM8, TMEM126A, TRAPPC12, TSFM, TUBB4A, UCHL1, VPS33A, WARS2, WDR36, WDR73, WFS1, YME1L1,*and* ZNHIT3.*

The capture panel was designed with the SureDesign of Agilent Technologies, including exons and exons/introns boundaries. Libraries were constructed by using SureSelect DNA target enrichment probes according to the manufacturer’s recommendations (Agilent Technologies France, les Ullis, France). gDNA samples were fragmented using Agilent SureSelect Enzymatic Fragmentation Kit Library. After molecular adaptors and barcode ligation, libraries were amplified and hybridized with target-specific probes of the gene panel. The probe/DNA complexes were captured with streptavidin-coated beads and amplified. Final library concentrations were determined using the Qubit dsDNA High Sensitivity Assay Kit (Thermo Fisher Scientific, Waltham, MA, USA). Twelve libraries were pooled in equimolar concentration (150 pM) and sequenced on an Ion Proton apparatus (Ion Torrent technology, Thermo Fisher Scientific, Waltham, MA, USA).

Variant calling, annotation, and prioritization of genetic variants were performed using our in-house pipeline. Variant calling was based on three variant callers: deepvariant, strelka and gatkHC. Annotation and prioritization steps were performed using the NCBI variant reporter and ANNOVAR. Classification of nuclear gene variants was determined using the Varsome [19] and Franklin algorithms [20] based on the standards and guidelines of the American College of Medical Genetics and Genomics (ACMG) [21]. Variants consistent with the clinical presentation and the mode of inheritance of classes 5 (Pathogenic), 4 (Probably pathogenic), and 3 (VUS) strongly predicted as pathogenic by the algorithms were retained. The involvement of pathogenic mtDNA variants was determined using the Mitomap database [22].

### Statistical analyses

Comparisons between the two patient groups (affected vs non-affected) were performed using a Wilcoxon rank sum test with continuity correction in R version 4.3.0 (R Foundation for Statistical Computing, Vienna, Austria; https://www.R-project.org/). Only measurements of the right eyes were included in the statistical OCT analysis. Adjustment for age, sex, drug use, smoking status, and duration of alcohol intoxication was performed in the multivariate analysis using Statistical Analysis System version 9.4 (SAS Institute Inc., Cary, NC, USA). A *p*-value (*p*) of less than 0.05 was considered statistically significant.

### Data availability

Anonymized data from this study are available from the corresponding author, upon reasonable request.

## Results

### Characteristics of the studied population

The general and addictological characteristics of the 102 patients at inclusion are summarized in Table 1. The mean age of the patients was 45.3 years (21.5–62.3).

**Table 1:** General and addictological characteristic of the studied population

		Patients (N = 102)
Sex	Male	80 (78.4%)
	Female	22 (21.6%)
Family history	Alcohol use disorder	51 (50.0%)
Alcohol-related systemic dysfunction	None	86 (84.3%)
	Peripheral neuropathy	9 (8.8%)
	Hepatic cirrhosis	7 (6.9%)
Folate supplementation at inclusion	None	40 (39.2%)
	Oral	36 (35.3%)
	Intravenous	3 (2.9%)
	Intramuscular	1 (1.0%)
	Missing	22 (21.6%)
Addictological characteristics of the cohort		
Ongoing daily alcohol intake (in grams per day)	< 150	45 (44.6%)
	151–300	32 (31.7%)
	> 300	24 (23.8%)
	Not reported by the patients	1 (1.0%)
Duration of alcohol intoxication (in years)	< 10	58 (58.6%)
	11–20	26 (26.3%)
	> 20	15 (15.2%)
	Not reported by the patients	3 (3.0%)
Severity of alcohol intoxication according to the DSM-5	Light	3 (3.0%)
	Medium	13 (12.9%)
	Severe	85 (84.2%)
	Not evaluable	1 (1.0%)
Tobacco consumption (in packets per year)	< 10	25 (24.5%)
	10–19	20 (19.6%)
	> 20	57 (55.9%)
Duration of smoking (in years)	< 15	28 (27.5%)
	15–29	31 (30.4%)
	> 30	43 (42.2%)
Drug use	None	52 (51.0%)
	Cannabis	20 (19.6%)
	Cocaine	14 (13.7%)
	Heroin	9 (8.8%)
	Other drugs	7 (6.9%)

### Optic nerve features, measured with OCT

**Fig. 1:**
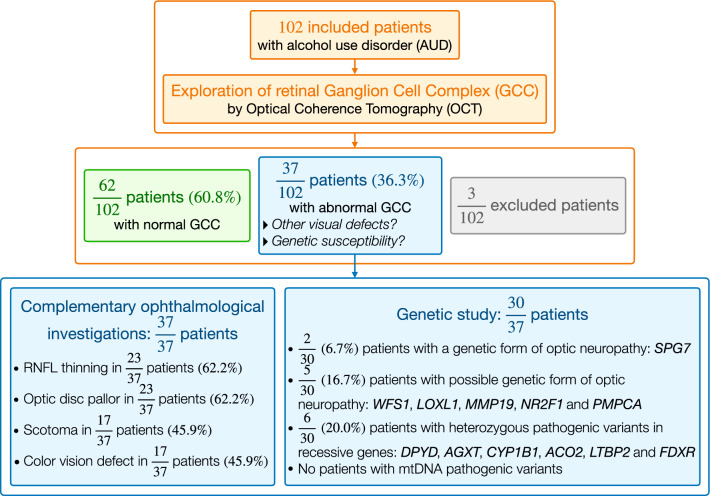
Workflow chart and main findings

Of the 102 initially included patients, three (2.9%) patients were excluded due to artefacts on the OCT imaging. Among the 99 remaining patients, 37 (37.3%) displayed thinning of the GCC layer in at least one eye (affected group), compared to the 62 (62.6%) unaffected patients, with normal GCC thickness values (*p* < 1.10^–3^) (Fig. 1). When 37 affected patients were compared with the 62 unaffected patients, the affected group showed a significant thinning of all the other internal retinal structures, measured arbitrarily on the right eye, as follows: mean thickness of the ganglion cell layer (GCL) 55.7 ± 6.9 μm *vs* 65.4 ± 5.8 μm (*p* = 2.10^–9^); mean thickness of the RNFL 89.1 ± 12.4 μm *vs* 107.3 ± 9.8 μm (*p* = 2.10^–10^) and mean thickness of GCL + inner plexiform layer 88.7 ± 9.5 μm *vs* 105.8 ± 7.7 μm (*p* = 4.10^–13^). Compared to the group with no GCC thinning (10.1 ± 8.2 years), the group with GCC thinning had a significantly longer history of alcohol consumption (16.1 ± 12.9 years, *p* = 0.016). There were no differences between the two groups in terms of quantity of alcohol (in grams per day) and tobacco (in packs per year) consumed.

**Fig. 2:**
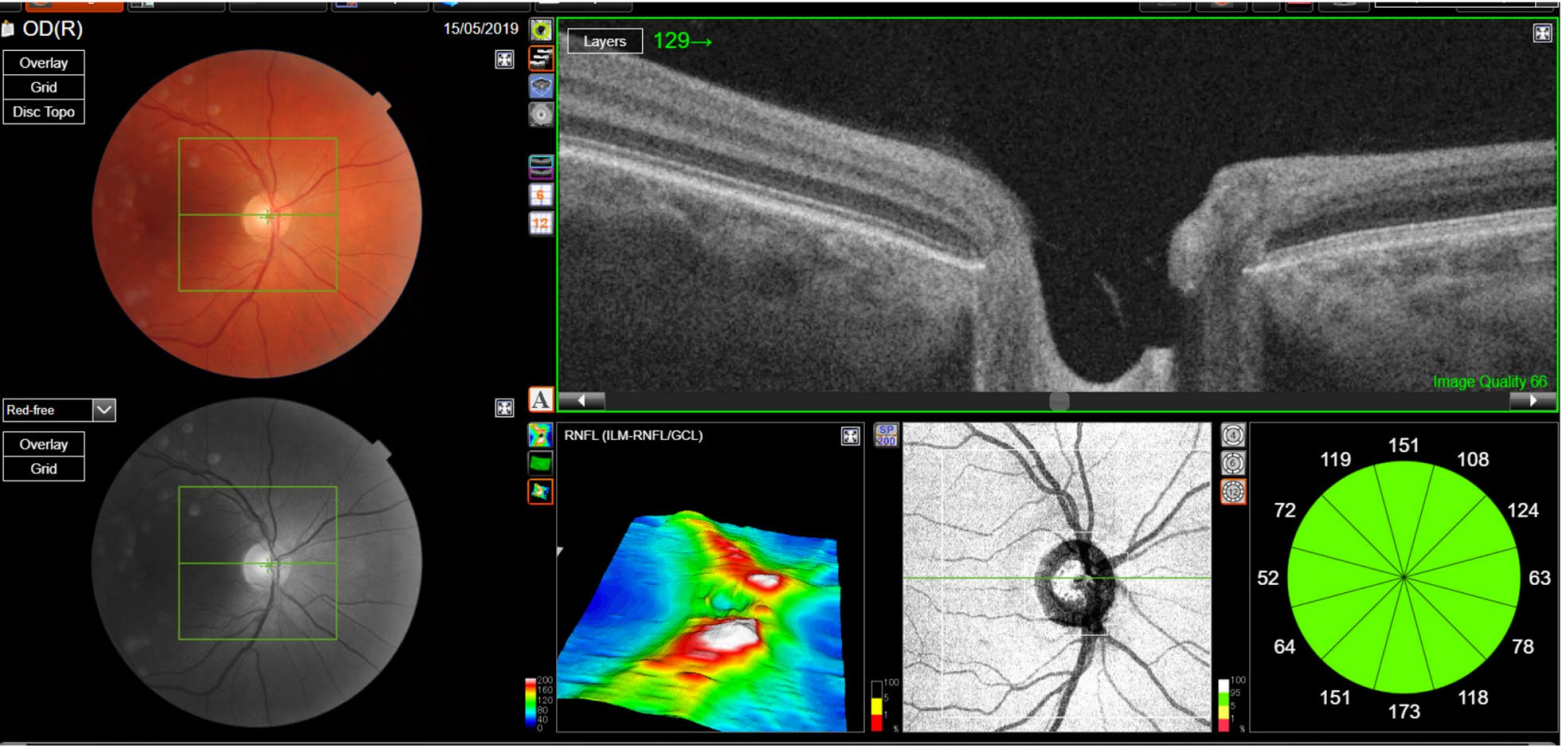
Retinal imaging in a normal patient, showing a normal appearance of the right optic nerve head on a colour photograph (upper left corner). Optical coherence tomography (OCT) images show a cross sectional analysis or the peripapillary retinal nerve fiber layer (RNFL) thickness, compared to a normative database

**Fig. 3:**
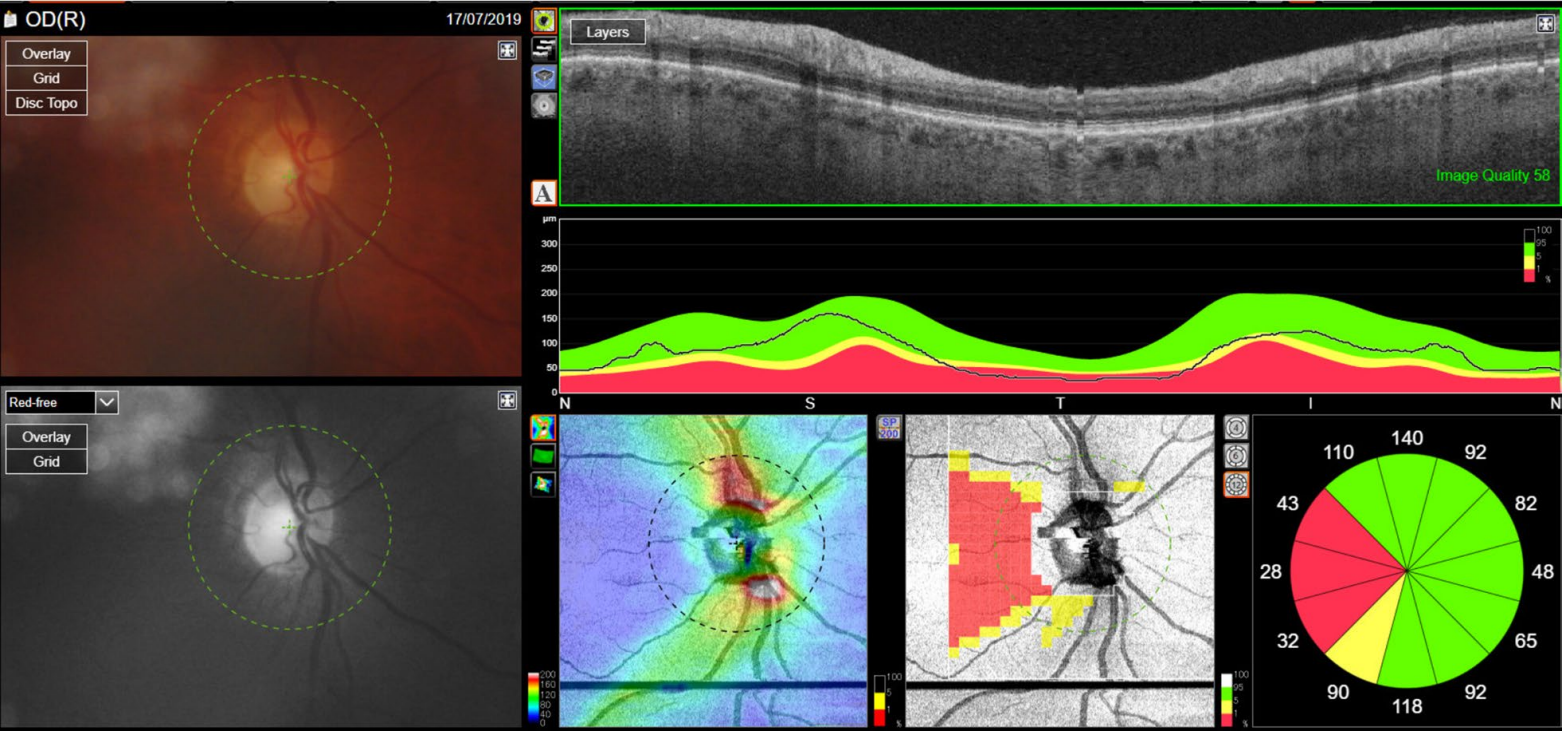
Retinal and optic nerve imaging of the right eye in patient 24, carrying the heterozygous variant NM_002429:c.173 + 1G>A (splicing variant) in the *MMP19* gene. The standard retinal photograph is within normal limits (upper left corner), but OCT shows an optic neuropathy and RNFL loss affecting the papillo-macular bundle (decreased RNFL values, in the red colored areas)

Clinically, among the 37 patients with abnormal OCT findings, 23 patients (62%) displayed fundoscopically optic disc pallor, 17 (46%) patients had a central or caeco-central scotoma on Goldman visual fields and 17 patients (46%) had altered color vision. Representative OCT images of one patient without optic atrophy and another patient with optic atrophy and genetic mutation are shown in Figs. 2 and 3.

### Vitamin status

Among the 72 patients whose plasma vitamin levels were measured at admission, 23 patients (32%) were deficient in vitamin B9 (folate), but none of them presented a deficiency in vitamins B1, B6 or B12. Folate deficiency was present in nine patients with GCC thinning and in 14 patients with unaltered GCC thickness, suggesting that folate deficiency was not significantly associated with GCC alteration (*p* = 0.27). There was no association with folate deficiency after multivariate adjustment for age, sex and drug use. There was no correlation between the thinning of GCC and the severity of alcohol intoxication (p = 0.11), but the involvement of the GCC was significantly more pronounced depending on the duration of alcohol intoxication (p = 0.02).

### Genetic susceptibility

Among the 37 patients in the group with retinal neuronal loss, 30 underwent a genetic study. Their data, summarized in Table 2, are presented in detail in Supplementary Table 1. Among these 30 patients, two disclosed (2/30, 6.7%) a Pathogenic variant in the *SPG7* gene. Five other patients (5/30, 16.7%) were affected by variants of uncertain significance (VUS) presenting arguments in favor of their pathogenicity in the *WFS1*, *LOXL1*, *MMP19*, *NR2F1* and *PMPCA* genes. In the same group, 20.0% (6/30) of patients carried heterozygous deleterious variants in recessive genes (*DPYD*, *AGXT*, *CYP1B1*, ACO2, *LTBP2* and *FDXR*). All the identified variants were in nuclear genes and no patient had pathogenic genetic variants in mitochondrial DNA. Taken together, a total of 23.3% (7/30) of patients who underwent genetic testing, had definite or possible genetic susceptibility. Among patients with optic neuropathy, there was no significant difference in the severity of visual impairment (presence or absence of papillary pallor and thickness of the RNFL layer) between those carrying an identified variant and those without.

**Table 2:** Overview of the results of genetic analysis in patients with retinal ganglion cell involvement

Individual #	Sex	Age	Findings	Gene and DNA variant
1	M	33	Heterozygous variant in a recessive gene	***DPYD***, Heterozygous c.1905 + 1G>A (p.?), Intron 14 of 22
4	F	51	Heterozygous variant in a recessive gene	***AGXT***, Heterozygous c.697C>T (p.Arg233Cys), Exon 7 of 11
**6**	F	36	**Positive**—Possible genetic form of optic neuropathy (*WFS1*), and heterozygous variant in a recessive gene	***WFS1***, Heterozygous c.2072G>T (p.Ser691Ile), Exon 8 of 8
				***L2HGDH***, Heterozygous c.1004G>A (p.Arg335Gln), Exon 8 of 10
8	M	28	Heterozygous variant in a recessive gene	***CYP1B1***, Heterozygous c.1147G>A (p.Ala383Thr), Exon 3 of 3
12	M	39	Heterozygous variant reported in recessive condition	***ACO2***, Heterozygous c.220C>G (p.Leu74Val), Exon 3 of 18
**14**	M	57	**Positive**—Possible genetic form of optic neuropathy	***LOXL1***, Heterozygous c.1468G>A (p.Gly490Ser), Exon 4 of 7
**17**	F	55	**Positive**—Genetic form of optic neuropathy	***SPG7***, Heterozygous c.1048C>A (p.Pro350Thr), Exon 8 of 10
**19**	M	52	**Positive**—Genetic form of optic neuropathy (SPG7), and heterozygous variant in a recessive gene	***SPG7***, Heterozygous c.1408C>T (p.Arg470*), Exon 10 of 17
				***ALG3***, Heterozygous c.778C>T (p.Arg260Cys), Exon 6 of 9
**24**	M	47	**Positive**—Possible genetic form of optic neuropathy	***MMP19***, Heterozygous c.173 + 1G>A (p.?), Intron 2 of 8
**27**	M	57	**Positive**—Possible genetic form of optic neuropathy	***NR2F1***, Heterozygous c.883T>C (p.Phe295Leu), Exon 2 of 3
30	M	28	Heterozygous variant in a recessive gene	***LTBP2***, Heterozygous c.4964A>G (p.Tyr1655Cys), Exon 34 of 36
**31**	M	40	**Positive**—Possible genetic form of optic neuropathy	***PMPCA***, Heterozygous c.1111C>A (p.His371Asn), Exon 10 of 13
35	M	52	Heterozygous variant in a recessive gene	***FDXR***, Heterozygous c.916C>T (p.Arg306Cys), Exon 9 of 12
*2, 3, 5, 7, 9, 11, 13, 15, 16, 20, 21, 22, 25, 26, 28, 32, 34*	15 M/2 F	23–63	**Negative**—Established or likely causes of optic neuropathy not identified	No established or probable variants were identified in 17 individuals

Among 37 patients with ganglion cell complex involvement, genetic analysis was performed in 30 of them. Optic neuropathies of genetic origin (n = 2/30; 6.7%, both in *SPG7*). Possible optic neuropathy of genetic origin (n = 5/30; 16.7%, *WFS1*, *LOXL1*, *NR2F1*, *MMP19* and *PMPCA*). This represents a total of 23.3% of patients with possible genetic susceptibility (7/30; individual # in bold). Genetic susceptibility conferred by a heterozygous variant in a recessive gene (n = 6/30; 20.0%, *DPYD*, *AGXT*, *CYP1B1*, *ACO2*, *LTBP2* and *FDXR*)? No genetic susceptibility factors identified (n = 17/30, 56.7%; individual # in italics). The complete results are provided in Supplementary Table 1

The first case was a 36-year-old female patient (# 6 in Table 2 and Supplementary Table 1) carrying a heterozygous missense variant c.2072G>T, with unknown significance (VUS) close to Likely Pathogenic classification and with high predictive pathogenicity scores, in the *WFS1* gene. This gene (Wolframin, chromosomal location: 4p16.1, eight exons) encodes a cation-selective ion channel transmembrane protein, located in the endoplasmic reticulum and ubiquitously expressed. It is involved in recessive Wolfram and dominant Wolfram-like syndromes. Given that the transmission being recessive or dominant, this variant alone could explain the appearance of an optic neuropathy. We did not found any information on this variant in the literature. This patient also carried a heterozygous missense variant c.1004G>A, VUS close to Likely Pathogenic and not previously reported in the literature, in the *L2HGDH* gene. This gene (l-2-hydroxyglutarate dehydrogenase, chromosomal location: 14q21.3, 10 exons) encodes a mitochondrial flavin adenine dinucleotide (FAD)-dependent enzyme that oxidizes l-2-hydroxyglutarate to alpha-ketoglutarate. Mutations in this gene cause l-2-hydroxyglutaric aciduria, a recessive neurometabolic disorder resulting in mental retardation sometimes associated with optic atrophy. This variant was also classified VUS close to Likely Pathogenic in this syndrome. As this genetic disease is recessive, this variant can only confer susceptibility to optic neuropathy but it could act synergistically with the *WFS1* variant to increase susceptibility, to optic nerve damage.

The second case (# 14) was a 57-year-old man with a heterozygous missense variant c.1468G>A in the *LOXL1* gene, not previously reported in the literature. This gene (Lysyl oxidase homolog 1, chromosomal location: 15q24.1, seven exons) encodes a member of the lysyl oxidase gene family involved in the biogenesis of connective tissue. It is associated with susceptibility for exfoliation syndrome, a common age-related disorder of the extracellular matrix frequently associated with secondary open-angle glaucoma. This variant was classified as VUS with both high conservation and pathogenicity scores. As the transmission is dominant, this variant could by itself explain the optic nerve vulnerability. Clinically, there was no evidence of pseudoexfoliation syndrome predisposing the patient to a glaucomatous optic neuropathy. We did not find any data indicating whether mutations in this gene could be associated with other hereditary optic neuropathies not related to the exfoliative syndrome.

The third case (# 17) was a 55-year-old woman carrying a heterozygous missense variant c.1048C>A, not reported in the literature, in the *SPG7* gene. This gene (Paraplegin, chromosomal location: 16q24.3, 17 exons) encodes a transmembrane metalloprotease that is a member of the AAA protein family located to the inner mitochondrial membrane. It is involved in spastic paraplegia and possibly isolated optic neuropathy. This variant was classified as Pathogenic with a high interspecies conservation of the amino acid. As this genetic disorder is recessive or dominant, this variant could by itself explain the optic neuropathy.

The fourth case (# 19) was a 52-year-old man who also carried a heterozygote nonsense variant c.1408C>T, not previously reported, in the *SPG7* gene. This variant was classified as Pathogenic. As the genetic disorder is recessive or dominant, this variant could by itself explain the optic neuropathy. This patient also carried a heterozygous missense variant c.778C>T, not previously reported, in the *ALG3* gene. This gene (Dolichyl-P-Man:Man(5)GlcNAc(2)-PP-dolichyl mannosyltransferase, chromosomal location: 3q27.1, nine exons) encodes an enzyme involved in glycosylation. It is involved in congenital disorder of glycosylation that has been associated with optic neuropathy. This variant was classified as VUS but close to Likely Pathogenic in this syndrome with a high interspecies conservation of the amino acid and a low frequency in population. As this genetic disease is recessive, this variant can only confer susceptibility to optic neuropathy, but it could act synergistically with the *SPG7* variant to increase susceptibility to optic nerve damage.

The fifth case (# 24) was a 47-year-old male carrying a heterozygous splicing variant c.173 + 1G>A, not previously reported, in the *MMP19* gene. This gene (Matrix metalloproteinase-19, chromosomal location: 12q13.2, eight exons) is a metalloproteinase involved in the breakdown of extracellular matrix. It is involved in cavitary optic disc anomalies that can lead to optic neuropathy. This variant was classified as VUS but close to Likely Pathogenic with a high interspecies conservation of the amino acid and a high relative pathogenicity. As this genetic disorder is dominant, this variant may be directly responsible for optic neuropathy. It should be noted that this patient also suffers from peripheral neuropathy without it being possible to know whether this was related to alcohol toxicity, to genetic susceptibility, or both. Indeed, there are several examples of overlap between optic and peripheral inherited neuropathies, as, for example, in the case of *MFN2* or *SLC24A46* genes.

The sixth case (# 27) was a 57-year-old man carrying a heterozygous missense variant c.883T>C, not previously reported, in the *NR2F1* gene. This gene (Nuclear Receptor subfamily 2, group F, member 1, chromosomal location 5q15, three exons) encodes a member of nuclear hormone receptor family. It is involved in the Bosch-Boonstra-Schaaf optic atrophy syndrome. This variant was classified as VUS but close to Likely Pathogenic with a high interspecies conservation of the amino acid, a high relative pathogenicity and has never been reported been found in the general population. As this genetic disorder is dominant, this variant may be directly responsible for optic neuropathy.

The seventh case (# 31) was a 40-year-old man carrying a heterozygous missense variant c.1111C>A in the *PMPCA* gene. This gene (Mitochondrial-processing peptidase subunit alpha, chromosomal location 9q34.3, 13 exons) encodes a peptidase located in the mitochondrial matrix. It is involved in spinocerebellar ataxia, autosomal recessive 2 that can lead to recessive or dominant optic neuropathy. This variant was classified as VUS but has never been found in the general population and has recently been reported to be associated with dominant transmission of late-onset optic neuropathy [23]. This recent report supports that this variant may be directly responsible for optic neuropathy.

The susceptibility variants identified in the other six patients were not firmly responsible on their own for a genetic form of optic neuropathy since they were found in recessives genes (*DPYD*, ACO2, *AGXT*, *CYP1B1*, *LTBP2* and *FDXR*) with a single affected allele.

## Discussion

The two main findings of our study are: (1) patients with AUD are frequently affected by asymptomatic structural damage of the optic nerve, identified with OCT and (2) in patients with optic neuropathy associated with AUD, there is a high prevalence of genetic variants, suggesting a genetic susceptibility to optic neuropathy. Given the debate surrounding the pathophysiology of this clinical entity, systematic OCT screening could be helpful in identifying asymptomatic AUD patients with retinal neuronal loss. Indeed, OCT is a sensitive method, allowing early and objective detection of optic neuropathies, via loss of RNFL and/or GCC thickness [24], as has been demonstrated in early glaucoma [25, 26] and hereditary optic neuropathies [27]. The prevalence of optic neuropathy in AUD is not well known; it has been hypothesized that 10% of AUD patients are clinically affected by a toxic optic neuropathy [28].

Among the 30 patients with GCC thinning who underwent a genetic analysis, seven patients carried pathogenic genetic variants that could account for optic neuropathy, in the absence of alcohol intoxication. These patients may therefore be considered to carry a true genetic background of hereditary optic neuropathy, possibly enhanced by excessive alcohol consumption. This surprising result means that at least 6.9% of the patients in the initial cohort (7/102) and 23.3% of those with GCC damage on OCT (7/30) could be carriers of a true genetic optic neuropathy. All variants identified in these patients were found in six dominant genes (*WFS1*, *LOXL1*, *SPG7*, *MMP19*, *NR2F1* and *PMPCA*). Several of these genes, such as *WFS1*, *SPG7*, and *NR2F1*, are common causes of hereditary optic neuropathies and none of them has been previously associated with alcohol susceptibility, to the best of our knowledge. Since dominant genes are often associated with incomplete penetrance, alcohol could be a penetrance factor in this context. A recent study of 97 patients with optic neuropathies explored through a panel of 22 sequenced genes identified a genetic cause in 20.2% and suggested that genetic variants were more prevalent in patients who reported excessive alcohol use [29].

In addition to these patients with possible inherited optic neuropathies, six other patients (20.0% of patients with an alteration of the GCC) carried suspected unique pathogenic variants in six recessive genes (*DPYD*, *AGXT*, *CYP1B1*, *ACO2*, *LTBP2* and *FDXR*). Many genes responsible for hereditary optic neuropathies present a double dominant and recessive transmission, such as *OPA1*, *ACO2*, *MFN2* and *SPG7*, for example, it is thus tempting to speculate that when only one allele is affected in a recessive form it could nevertheless contribute to the genetic susceptibility. These variants could provide a moderate genetic susceptibility by weakening retinal ganglion cells. On their own, they could not cause optic neuropathy, but they could act synergistically with alcohol to do so.

All the mutations in our study affected nuclear genes and none of the patients carried pathogenic mitochondrial DNA variants. This is not surprising, since our study did not include patients with acute visual loss, which is a hallmark of LHON. Conversely, patients with nuclear gene mutations commonly display slow progression, sometimes being totally asymptomatic, much like the patients included in this study.

In our previous study [17], we screened 2186 patients with suspicion of hereditary optic neuropathy, analyzing them using our sequencing panel of 87 nuclear genes. The percentage of patients carrying pathogenic variants in these nuclear genes was 27%, which is quite close to the 23% positive cases we found here (7 out of the 30 patients with optic neuropathy). This indicates that even though these optic neuropathies were unmasked by alcohol intoxication, they nonetheless remain genuine optic neuropathies deserving genetic exploration. It is also noteworthy that 4 out of the 7 cases identified here with genetic variants, showed dominant mutations in the *SPG7*, *WFS1*, and *NR2F1* genes, which were among the top 10 genes most frequently identified in our previous study, further strengthening the proximity between these common and alcohol-related optic neuropathies.

If our results were confirmed by other studies, it could significantly alter the management of patients with AUD. Besides demonstrating that systematic screening for optic neuropathy using OCT allows for early detection of optic neuropathies, our study also shows that these "toxico-nutritional" optic neuropathies warrant genuine genetic management. For patients with genetic variants, genetic counseling would become essential to detect other familial cases, potentially offer prenatal diagnosis for couples planning to have children, and most importantly, to induce prevention of retinal ganglion cell damage by cessation of alcohol intoxication. This prevention could be further motivated by the fact that optic neuropathy is documented by OCT and its genetic origin.

From a mechanistic standpoint, the six genes implicated in the dominant forms of optic neuropathies identified here do not point to a common pathogenic mechanism. Indeed, the implicated genes are involved in mitochondrial metabolism for two of them (*SPG7* and *PMPCA*), endoplasmic reticulum function for another gene (*WFS1*), extracellular tissue metabolism for two of them (*LOXL1* and *MMP19*), and gene expression for the last one (*NR2F1*). Nevertheless, it is noteworthy that mitochondrial and endoplasmic reticulum impairments, as well as the interaction between these two organelles at the level of mitochondrial-associated membranes, are a frequent mechanism of neurodegeneration and optic neuropathies, particularly through the protein and oxidative stresses they generate, as shown for glaucoma [30]. To our knowledge, no link has been previously established between these six genes and alcohol-induced retinal ganglion cells neurotoxicity.

## Conclusion

Our study suggests that a third of patients with AUD may suffer from retinal neuronal and axonal loss causing asymptomatic optic neuropathies, easily detectable using OCT. Furthermore, a quarter of patients with optic neuropathy associated with AUD have a genetic predisposition towards optic nerve damage. Future work, undertaken by specialists in addictology, ophthalmology, and molecular genetics might determine whether it would be useful systematically screen patients with AUD to detect asymptomatic optic neuropathy and genetic disease susceptibility.

### Supplementary Information

Supplementary Material 1: Supplementary Table 1: Results of genetic analysis in patients with retinal ganglion cell involvement. Among 37 patients with ganglion cell complex involvement, genetic analysis was performed in 30 of them. Optic neuropathies of genetic origin (n = 2/30; 6.7 %, SPG7). Possible optic neuropathy of genetic origin (n = 5/30; 16.7%, WFS1, LOXL1, NR2F1, MMP19 and PMPCA). This represents a total of 23.3% patients with possible genetic susceptibility (7/30). Genetic susceptibility conferred by a heterozygous variant in a recessive gene (n = 6/30; 20.0%, DPYD, AGXT, CYP1B1, ACO2, LTBP2 and FDXR)? No genetic susceptibility factors identified (n = 17/30, 56.7%). The phyloP scores represent as the log (P-value) under a null hypothesis of neutral evolution and can indicate both accelerated evolution as well as evolutionary conservation [31]: positive phyloP scores, indicating conservation; negative phyloP scores, indicating fast-evolving. Combined Annotation Dependent Depletion score (C-score) is a PHRED-like (− 10*log10(rank/total)) scaled score ranking a variant relative to all possible substitutions of the human genome (8.6 x 10^9^) [32]: a scaled C-score of greater of equal 10 indicates that these are predicted to be the 10% most deleterious substitutions that you can do to the human genome; a score of greater or equal 20 indicates the 1% most deleterious and so on. VUS: variant of uncertain significance. Variants class: variants were classed into class 3 (VUS), class 4 (LP, likely pathogenic) and class 5 (P, pathogenic) according to the ACMG criteria for variant classification [21].

## Data Availability

Not applicable.

## Abbreviations

ACMG: American college of medical genetics and genomics
AUD: Alcohol use disorder
CGL: Ganglion cell layer
DSM: Diagnostic and statistical manual of mental disorders
GCC: Ganglion cell complex
LHON: Leber hereditary optic neuropathy
mtDNA: Mitochondrial DNA
OCT: Optical coherence tomography
RNFL: Retinal nerve fiber layer
VUS: Variants of uncertain significance

## References

[1] Horner JF (1878). Ueber intoxicationsamblyopie. Corresp Schweiz Aerzte.

[2] Grzybowski A, Graham EH (2011). Tobacco optic neuropathy (TON)—the historical and present concept of the disease. Acta Ophthalmol.

[3] Grzybowski A, Pieniążek M (2014). Tobacco-alcohol amblyopia does not exist. Acta Ophthalmol.

[4] Lessell S (1998). Nutritional amblyopia. J Neuroophthalmol.

[5] Milea D, Cassoux N, LeHoang P (2000). Blindness in a strict vegan. N Engl J Med.

[6] The Cuba Neuropathy Field Investigation Team (1995). Epidemic optic neuropathy in Cuba clinical characterization and risk factors. New Engl J Med.

[7] Sadun AA, Martone JF (1995). Cuba: response of medical science to a crisis of optic and peripheral neuropathy. Int Ophthalmol.

[8] Syed S, Lioutas V (2013). Tobacco-alcohol amblyopia: a diagnosis dilemma. J Neurol Sci.

[9] González-Quevedo A, Santiesteban-Frexas R, Eells JT, Lima L, Sadun AA (2018). Cuban epidemic neuropathy: insights into the toxic-nutritional hypothesis through international collaboration. MEDICC Rev.

[10] Xie X, Feng K, Wang J, Zhang M, Hong J, Zhang H (2022). Comprehensive visual electrophysiological measurements discover crucial changes caused by alcohol addiction in humans: clinical values in early prevention of alcoholic vision decline. Front Neural Circuits.

[11] Sanz-Morello B, Ahmadi H, Vohra R, Saruhanian S, Freude KK, Hamann S (2021). Oxidative stress in optic neuropathies. Antioxidants (Basel).

[12] Kirkman MA, Yu-Wai-Man P, Korsten A, Leonhardt M, Dimitriadis K, De Coo IF (2009). Gene-environment interactions in Leber hereditary optic neuropathy. Brain.

[13] Rabenstein A, Catarino CB, Rampeltshammer V, Schindler D, Gallenmüller C, Priglinger C (2021). Smoking and alcohol, health-related quality of life and psychiatric comorbidities in Leber's Hereditary Optic Neuropathy mutation carriers: a prospective cohort study. Orphanet J Rare Dis.

[14] Korkiamäki P, Kervinen M, Karjalainen K, Majamaa K, Uusimaa J, Remes AM (2013). Prevalence of the primary LHON mutations in Northern Finland associated with bilateral optic atrophy and tobacco-alcohol amblyopia. Acta Ophthalmol.

[15] Cullom ME, Heher KL, Miller NR, Savino PJ, Johns DR (1993). Leber's hereditary optic neuropathy masquerading as tobacco-alcohol amblyopia. Arch Ophthalmol.

[16] Zaslavsky K, Margolin EA (2021). Leber's hereditary optic neuropathy in older individuals because of increased alcohol consumption during the COVID-19 pandemic. J Neuroophthalmol.

[17] Rocatcher A, Desquiret-Dumas V, Charif M, Ferré M, Gohier P, Mirebeau-Prunier D (2023). The top 10 most frequently involved genes in hereditary optic neuropathies in 2186 probands. Brain.

[18] Boucret L, Bris C, Seegers V, Goudenège D, Desquiret-Dumas V, Domin-Bernhard M (2017). Deep sequencing shows that oocytes are not prone to accumulate mtDNA heteroplasmic mutations during ovarian ageing. Hum Reprod.

[19] Kopanos C, Tsiolkas V, Kouris A, Chapple CE, Albarca AM, Meyer R (2019). VarSome: the human genomic variant search engine. Bioinformatics.

[20] https://franklin.genoox.com. Version 65.1. Date accessed: June 19, 2023.

[21] Richards S, Aziz N, Bale S, Bick D, Das S, Gastier-Foster J (2015). Standards and guidelines for the interpretation of sequence variants: a joint consensus recommendation of the American College of Medical Genetics and Genomics and the Association for Molecular Pathology. Genet Med.

[22] Lott MT, Leipzig JN, Derbeneva O, Xie HM, Chalkia D, Sarmady M (2013). mtDNA variation and analysis using mitomap and mitomaster. Curr Protoc Bioinformatics.

[23] Charif M, Chevrollier A, Gueguen N, Kane S, Bris C, Goudenège D (2022). Next-generation sequencing identifies novel PMPCA variants in patients with late-onset dominant optic atrophy. Genes (Basel).

[24] Biousse V, Danesh-Meyer HV, Saindane AM, Lamirel C, Newman NJ (2022). Imaging of the optic nerve: technological advances and future prospects. Lancet Neurol.

[25] Kim HJ, Jeoung JW, Yoo BW, Kim HC, Park KH (2017). Patterns of glaucoma progression in retinal nerve fiber and macular ganglion cell-inner plexiform layer in spectral-domain optical coherence tomography. Jpn J Ophthalmol.

[26] Shin JW, Sung KR, Park SW (2018). Patterns of progressive ganglion cell-inner plexiform layer thinning in glaucoma detected by OCT. Ophthalmology.

[27] Rönnbäck C, Milea D, Larsen M (2013). Imaging of the macula indicates early completion of structural deficit in autosomal-dominant optic atrophy. Ophthalmology.

[28] Donnadieu-Rigole H, Daien V, Blanc D, Michau S, Villain M, Nalpas B (2014). The prevalence of optic neuropathy in alcoholic patients, a pilot study. Alcohol Clin Exp Res.

[29] Chen AT, Brady L, Bulman DE, Sundaram ANE, Rodriguez AR, Margolin E (2019). An evaluation of genetic causes and environmental risks for bilateral optic atrophy. PLoS ONE.

[30] Pham JH, Stankowska DL (2023). Mitochondria-associated endoplasmic reticulum membranes (MAMs) and their role in glaucomatous retinal ganglion cell degeneration—a mini review. Front Neurosci.

[31] Pollard KS, Hubisz MJ, Rosenbloom KR, Siepel A (2010). Detection of nonneutral substitution rates on mammalian phylogenies. Genome Res.

[32] Kircher M, Witten DM, Jain P (2014). A general framework for estimating the relative pathogenicity of human genetic variants. Nat Genet.

