# Supplementary material for: Genetic susceptibility to optic neuropathy in patients with alcohol use disorder
Source: J Transl Med. 2024 May 25;22:495. doi: 10.1186/s12967-024-05334-0 (PMC11127293; doi:10.1186/s12967-024-05334-0)
Supplement: Supplementary file 1 — Supplementary Material 1: Supplementary Table 1: Results of genetic analysis in patients with retinal ganglion cell involvement. Among 37 patients with ganglion cell complex involvement, genetic analysis was performed in 30 of them. Optic neuropathies of genetic origin (n = 2/30; 6.7 %, SPG7). Possible optic neuropathy of genetic origin (n = 5/30; 16.7%, WFS1, LOXL1, NR2F1, MMP19 and PMPCA). This represents a total of 23.3% patients with possible genetic susceptibility (7/30). Genetic susceptibility conferred by a heterozygous variant in a recessive gene (n = 6/30; 20.0%, DPYD, AGXT, CYP1B1, ACO2, LTBP2 and FDXR)? No genetic susceptibility factors identified (n = 17/30, 56.7%). The phyloP scores represent as the log (P-value) under a null hypothesis of neutral evolution and can indicate both accelerated evolution as well as evolutionary conservation [31]: positive phyloP scores, indicating conservation; negative phyloP scores, indicating fast-evolving. Combined Annotation Dependent Depletion score (C-score) is a PHRED-like (− 10*log10(rank/total)) scaled score ranking a variant relative to all possible substitutions of the human genome (8.6 x 10^9^) [32]: a scaled C-score of greater of equal 10 indicates that these are predicted to be the 10% most deleterious substitutions that you can do to the human genome; a score of greater or equal 20 indicates the 1% most deleterious and so on. VUS: variant of uncertain significance. Variants class: variants were classed into class 3 (VUS), class 4 (LP, likely pathogenic) and class 5 (P, pathogenic) according to the ACMG criteria for variant classification [21]. [file 12967_2024_5334_MOESM1_ESM.pdf]

Supplementary Table 1. Results of genetic analysis in patients with retinal ganglion cell involvement.

Ind. #	Sex	Age	Findings	Gene and DNA variant	Genomic DNA change (hg19)	cDNA Reference sequence	dbSNP rsID	Average frequency (gnomAD v2.1.1)	100 vertebrates basewise conservation (phyloP100wayAll from UCSC Genome)	Relative pathogenicity (CADD version 1.6 score)	ACMG Criteria for variant Classification	Variant Class	Interpretation summary
1	M	33	Heterozygous variant in a recessive gene	***DPYD***, Heterozygous c.1905+1G>A (p.?), Intron 14 of 22	chr1-97915614 C>T	NM_000110.4	rs3918290	0.569% (1,608/282,660 alleles; 9 HMZ individuals)	Very High (7.43)	Top 0.1% (33)	PVS1 Strong<br>PP5 Strong	Class 5 Pathogenic (recessive)	Heterozygous splicing variant c.1905+1G>A in the *DPYD* gene involved in Dihydropyrimidine dehydrogenase deficiency (MIM# 274270) which is a recessive neurological syndrome, with possible optic atrophy, appearing under the toxic effect of 5-Fluorouracil. It has already been reported as a proven Pathogenic variant in this syndrome by van Kuilenburg *et al.* (2018), Zhu *et al.* (2015) and Lieber *et al.* (2013).
2	M	46	Negative - Established or likely causes of optic neuropathy not identified										
3	M	23	Negative - Established or likely causes of optic neuropathy not identified										
4	F	51	Heterozygous variant in a recessive gene	***AGXT***, Heterozygous c.697C>T (p.Arg233Cys), Exon 7 of 11	chr2-241814542 C>T	NM_000030.3	rs121908526	0.006% (14/251,410)	Low (1.12)	Top 1% (24.2)	PM2 Moderate<br>PM5 Moderate<br>PP3 Supporting	Class 5 Pathogenic (recessive)	Heterozygous missence variant c.697C>T in the *AGXT* gene involved in Hyperoxaluria, primary, type 1 (MIM# 259900), characterized by an accumulation of calcium oxalate in various bodily tissues, that can lead to optic neuropathy. This variant is a proven Pathogenic variant in this syndrome.
5	M	54	Negative - Established or likely causes of optic neuropathy not identified										
6	F	36	Positive - Possible genetic form of optic neuropathy.	***WFS1***, Heterozygous c.2072G>T (p.Ser691Ile), Exon 8 of 8	chr4-6303594 G>T	NM_006005.3	rs1157038779	Variant not found	High (4.53)	Top 1% (27.7)	PM2 Moderate<br>PP3 Supporting<br>PM1 Moderate	Class 3 VUS/close to Likely pathogenic (dominant/recessive)	Heterozygous missense variant c.2072G>T in the *WFS1* gene involved in Wolfram (MIM# 222300) and Wolfram-like (MIM# 614296) syndromes, characterized by the association of diabetes mellitus, optic atrophy, diabetes insipidus and deafness. This variant is Classified as VUS but close to Likely pathogenic and the inheritance being recessive or dominant, it could explain alone the optic neuropathy.
			Heterozygous variant in a recessive gene	***L2HGDH***, Heterozygous c.1004G>A (p.Arg335Gln), Exon 8 of 10	chr14-50734531 C>T	NM_024884.3	rs779733566	0.003% (8/251,424)	Very High (7.64)	Top 0.1% (32)	PM2 Moderate<br>PP3 Moderate	Class 3 VUS/close to Likely pathogenic (recessive)	Heterozygous missense variant c.1004G>A in the *L2HGDH* gene involved in Hydroxyglutaric aciduria (MIM# 609584), a neurological metabolic syndrome that may lead to optic atrophy. This variant is a Classified as VUS but close to Likely pathogenic in this syndrome.
7	M	53	Negative - Established or likely causes of optic neuropathy not identified										
8	M	28	Heterozygous variant in a recessive gene	***CYP1B1***, Heterozygous c.1147G>A (p.Ala383Thr), Exon 3 of 3	chr2-38298350 C>T	NM_000104.4	Variant not found	Variant not found	Very High (5.85)	Top 1% (26.5)	PM2 Moderate<br>PP3 Supporting<br>PM1 Moderate	Class 3 VUS/close to Likely pathogenic (recessive)	Heterozygous missense variant c.1147G>A in the *CYP1B1* gene involved in Glaucoma 3A, primary open angle, congenital, juvenile, or adult onset (MIM# 231300). This variant is Classified as VUS but close to Likely pathogenic and has not previously been found in the population.
9	F	40	Negative - Established or likely causes of optic neuropathy not identified										
11	M	42	Negative - Established or likely causes of optic neuropathy not identified										
12	M	39	Heterozygous variant reported in recessive condition	***ACO2***, Heterozygous c.220C>G (p.Leu74Val), Exon 3 of 18	chr22-41903841 C>G	NM_001098.3	rs141772938	0.374% (1,055/282,256; 4 HMZ individuals)	Low (0.70)	Top 1% (23.2)	BP6 Supporting<br>BS1 Strong<br>BS2 Strong<br>PP2 Supporting<br>PP3 Supporting<br>PP5 Supporting	Class 3 VUS/close to Likely benign	Heterozygous missense variant c.220C>G in the *ACO2* gene that is a frequent cause of isolated or syndromic inherited Optic neuropathy, with both recessive et dominant inheritance (Optic atrophy 9; MIM# 616289). This variant is commonly Classified as VUS close to Likely benign but has been repeatedly published as Likely pathogenic with recessive inheritance (Metodiev *et al.*, 2014; Kelman *et al.*, 2018; Charif *et al.*, 2021),
13	M	56	Negative - Established or likely causes of optic neuropathy not identified										
14	M	57	Positive - Possible genetic form of optic neuropathy	***LOXL1***, Heterozygous c.1468G>A (p.Gly490Ser), Exon 4 of 7	chr15-74239526 G>A	NM_005576.4	rs754733212	0.001% (4/282,450)	Very High (9.11)	Top 0.1% (31)	PM2 Moderate<br>PP3 Supporting	Class 3 VUS	Heterozygous missense variant c.1468G>A in the *LOXL1* gene, associated with Susceptibility for exfoliation syndrome (MIM# 177650), that is a common age-related disorder of the extracellular matrix frequently associated with severe chronic secondary open-angle glaucoma and cataract. This variant is Classified as VUS with both high conservation and pathogenicity scores. The genetic disorder being dominant, this variant could alone explain the optic neuropathy.
15	M	54	Negative - Established or likely causes of optic neuropathy not identified										
16	M	58	Negative - Established or likely causes of optic neuropathy not identified										

**17**	F	55	Positive - Genetic form of optic neuropathy.	***SPG7*** , Heterozygous c.1048C>A (p.Pro350Thr), Exon 8 of 10	chr16-89598372 C>A	NM_003119.4	rs199789849	0.014% (39/281,528)	Very High (7.45)	Top 1% (24.4)	PM1 Moderate PM2 Moderate PM5 Supporting PP3 Supporting PP5 Strong	Class 5 Pathogenic (dominant/recessive)	Heterozygous missense variant c.1048C>A in the *SPG7* gene involved in Spastic paraplegia (MIM# 607259) and possibly optic neuropathy. This variant is Classified Pathogenic with a very high interspecies conservation of the amino acid. The genetic disorder being recessive or dominant, this variant could alone explain the optic neuropathy.
**19**	M	52	Positive - Genetic form of optic neuropathy.	***SPG7*** , Heterozygous c.1408C>T (p.Arg470*), Exon 10 of 17	chr16-89611139 C>T	NM_003119.4	rs748555510	0.001% (2/251,404)	Low (1.41)	Top 0.1% (39)	PVS1 Very Strong PM2 Moderate PP5 Supporting	Class 5 Pathogenic (dominant/recessive)	Heterozygous nonsense variant c.1408C>T in the *SPG7* gene involved in Spastic paraplegia (MIM# 607259) and possibly optic neuropathy. This variant is Classified as Pathogenic. The genetic disorder being recessive or dominant, this variant could alone explain the optic neuropathy.
			Heterozygous variant in a recessive gene	***ALG3*** , Heterozygous c.778C>T (p.Arg260Cys), Exon 6 of 9	chr3-183961733 G>A	NM_005787.6	rs749229743	0.005% (14/279,956)	Very High (6.56)	Top 1% (26.4)	PM2 Moderate PP3 Supporting	Class 3 VUS/close to Likely pathogenic (recessive)	Heterozygous missense variant c.778C>T in the *ALG3* gene involved in Congenital disorder of glycosylation (MIM# 601110) that can lead to optic neuropathy. This variant is Classified as VUS but close to Likely pathogenic in this syndrome with a very high interspecies conservation of the amino acid and a low frequency in population.
**20**	M	46	Negative - Established or likely causes of optic neuropathy not identified										
**21**	M	56	Negative - Established or likely causes of optic neuropathy not identified										
**22**	M	57	Negative - Established or likely causes of optic neuropathy not identified										
**24**	M	47	Positive. Possible genetic form of optic neuropathy	***MMP19*** , Heterozygous c.173+1G>A (p.?), Intron 2 of 8	chr12-56236136 C>T	NM_002429.6	rs145293054	0.082% (231/282,876)	High (4.77)	Top 0.1% (35)	PM2 Moderate PP3 Supporting	Class 3 VUS/close to Likely pathogenic (dominant)	Heterozygous splicing variant c.173+1G>A in the *MMP19* gene involved in Cavitary optic disc anomalies (MIM# 611543) that can lead to optic neuropathy. This variant is Classified as VUS but close to Likely pathogenic with a high interspecies conservation of the amino acid and a high relative pathogenicity. The genetic disorder being dominant, this variant may be directly responsible for optic neuropathy.
**25**	M	31	Negative - Established or likely causes of optic neuropathy not identified										
**26**	F	58	Negative - Established or likely causes of optic neuropathy not identified										
**27**	M	57	Positive - Possible genetic form of optic neuropathy	***NR2F1*** , Heterozygous c.883T>C (p.Phe295Leu), Exon 2 of 3	chr5-92924042 T>C	NM_005654.6	Variant not found	Variant not found	Very High (6.03)	Top 1% (26.3)	PM2 Moderate PP3 Supporting	Class 3 VUS	Heterozygous missense variant c.883T>C in the *NR2F1* gene involved in the Bosch-Boonstra-Schaaf optic atrophy syndrome (MIM# 615722). This variant is Classified as VUS with a high interspecies conservation of the amino acid, a high relative pathogenicity and has not previously been found in the population. The genetic disorder being dominant, this variant may be directly responsible for optic neuropathy.
**28**	M	34	Negative - Established or likely causes of optic neuropathy not identified										
**30**	M	28	Heterozygous variant in a recessive gene	***LTBP2*** , Heterozygous c.4964A>G (p.Tyr1655Cys), Exon 34 of 36	chr14-74969562 T>C	NM_000428.3	rs140493259	0.047% (132/282,278)	Very High (4.90)	Top 0.1% (32)	All criteria unmet	Class 3 VUS	Heterozygous missense variant c.4964A>G in the LTBP2 gene involved in recessive Glaucoma 3, primary congenital, D (MIM# 613086). This variant is Classified as VUS in this disease with high pathogenicity score.
**31**	M	40	Positive - Possible genetic form of optic neuropathy	***PMPCA*** , Heterozygous c.1111C>A (p.His371Asn), Exon 10 of 13	chr9-139313281 C>A	NM_015160.3	Variant not found	Variant not found	Moderate (3.72)	Top 1% (29.5)	PM2 Moderate PP3 Supporting	Class 3 VUS/close to Likely pathogenic (dominant)	Heterozygous missense variant c.1111C>A in the PMPCA gene involved in Spinocerebellar ataxia, autosomal recessive 2 (MIM# 213200) that can lead to recessive or dominant optic neuropathy. This variant is Classified as VUS but has not previously been found in the population and has recently been reported to be associated with dominant transmission of late-onset optic neuropathy (Charif et al 2022). The recently reported dominant transmission of late-onset optic neuropathy supports that this variant may be directly responsible for optic neuropathy.
**32**	M	63	Negative - Established or likely causes of optic neuropathy not identified										
**34**	M	58	Negative - Established or likely causes of optic neuropathy not identified										
**35**	M	52	Heterozygous variant in a recessive gene	***FDXR*** , Heterozygous c.916C>T (p.Arg306Cys), Exon 9 of 12	chr17-72860356 G>A	NM_024417.5	rs752143061	0.003% (7/241,968)	High (4.27)	Top 1% (24.7)	PM2 Moderate PP5 Strong	Class 4 Likely pathogenic (recessive)	Heterozygous missense variant c.916C>T in the FDXR gene involved in Auditory neuropathy and optic atrophy (MIM# 617717). This variant is Classified as Likely pathogenic in this syndrome.

Among 37 patients with ganglion cell complex involvement, genetic analysis was performed in 30 of them. Optic neuropathies of genetic origin (n = 2/30; 6.7 %, *SPG7*). Possible optic neuropathy of genetic origin (n = 5/30; 16.7%, *WFS1* , *LOXL1* , *NR2F1* , *MMP19* and *PMPCA*). This represents a total of 23.3% patients with possible genetic susceptibility (7/30). Genetic susceptibility conferred by a heterozygous variant in a recessive genes (n = 6/30; 20.0%, *DPYD* , *AGXT* , *CYP1B1* , *ACO2* , *LTBP2* and *FDXR*)? No genetic susceptibility factors identified (n = 17/30, 56.7%). The phyloP scores represent as the log (P-value) under a null hypothesis of neutral evolution and can indicate both accelerated evolution as well as evolutionary conservation [29]: positive phyloP scores, indicating conservation; negative phyloP scores, indicating fast-evolving. Combined Annotation Dependent Depletion score (C-score) is a PHRED-like (-10*log10(rank/total)) scaled score ranking a variant relative to all possible substitutions of the human genome (8.6x10^9) [30]: a scaled C-score of greater of equal 10 indicates that these are predicted to be the 10% most deleterious substitutions that you can do to the human genome; a score of greater or equal 20 indicates the 1% most deleterious and so on. VUS: variant of uncertain significance. Variants class: variants were classed into class 3 (VUS), class 4 (LP, likely pathogenic) and class 5 (P, pathogenic) according to the ACMG criteria for variant classification [20].
